# Dual Wavelength RP-HPLC Method for Simultaneous Determination of Two Antispasmodic Drugs: An Application in Pharmaceutical and Human Serum

**DOI:** 10.1155/2013/297285

**Published:** 2013-10-27

**Authors:** Najmul Hasan, Mathurot Chaiharn, Sauleha Khan, Hira Khalid, Nawab Sher, Farhan Ahmed Siddiqui, Muhammad Zain Siddiqui

**Affiliations:** ^1^Department of Microbiology, Faculty of Science, University of Karachi, Karachi 75270, Pakistan; ^2^Division of Biotechnology, Faculty of Science, Maejo University, Chiang Mai 50290, Thailand; ^3^Department of Chemistry, Faculty of Science, University of Karachi, Karachi 75270, Pakistan; ^4^Department of Chemistry, Government College University, Lahore 54000, Pakistan; ^5^Faculty of Pharmacy, Federal Urdu University of Science & Technology, Karachi 75300, Pakistan; ^6^Department of Chemistry, Faculty of Science, Federal Urdu University of Science & Technology, Karachi 75300, Pakistan

## Abstract

A reverse phase stability indicating HPLC method for simultaneous determination of two antispasmodic drugs in pharmaceutical parenteral dosage forms (injectable) and in serum has been developed and validated. Mobile phase ingredients consist of Acetonitrile : buffer : sulfuric acid 0.1 M (50 : 50 : 0.3 v/v/v), at flow rate 1.0 mL/min using a Hibar **μ**Bondapak ODS C_18_ column monitored at dual wavelength of 266 nm and 205 nm for phloroglucinol and trimethylphloroglucinol, respectively. The drugs were subjected to stress conditions of hydrolysis (oxidation, base, acid, and thermal degradation). Oxidation degraded the molecule drastically while there was not so much significant effect of other stress conditions. The calibration curve was linear with a correlation coefficient of 0.9999 and 0.9992 for PG and TMP, respectively. The drug recoveries fall in the range of 98.56% and 101.24% with 10 pg/mL and 33 pg/mL limit of detection and limit of quantification for both phloroglucinol and trimethylphloroglucinol. The method was validated in accordance with ICH guidelines and was applied successfully to quantify the amount of trimethylphloroglucinol and phloroglucinol in bulk, injectable form and physiological fluid. Forced degradation studies proved the stability indicating abilities of the method.

## 1. Introduction

**Figure 1:**
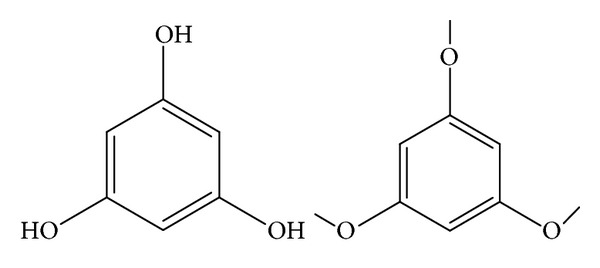
Chemical structures of phloroglucinol and trimethylphloroglucinol.

Chemically, phloroglucinol (1,3,5-trihydroxybenzene, PG) and its methylated derivative tri-O-methylphloroglucinol (TMP), Figure 1, are established pharmaceutical agents inhibit the action of catechol-O-methyl transferase, inducing relaxation of smooth muscles, and decreasing glycerol-induced abdominal pain and are also characterized by a swift and strong spasmolytic activity, hence relieving pain. Therefore, PG is often used in combination with trimethylphloroglucinol as an antispasmodic drug and is regarded to be effective in decreasing smooth muscle spasm. PG/TMP combination is recommended against biliary calculi, severe pain of urinary or gastrointestinal tract, pain of abdominal region, spastic conditions of the female genital system, and pain in gynecology [1–7].

Literature survey reveals that some of the analytical methods for phloroglucinol are available including extraction and high-performance liquid chromatography (HPLC) [8–10], HPLC-mass spectrometry [11, 12], gas chromatography-mass spectrometry [13], and spectrophotometry [14]. Other reported methods include titrimetry, spectrophotometry, paper chromatography, and flow injection analysis [15–20].

However, there is no simple and sensitive method to be followed on industrial basis especially in general quality control laboratories. Previously mentioned methods involve complicated instrumentation and serialization. Therefore, they cannot be followed in the laboratories particularly those of third world countries. Hence our group already developed a cost effective method for its fixed dose composition in tablet form [21], but still there was a need for an analytical method which would help to determine the active pharmaceutical ingredients (APIs) in parenteral products and physiological fluid. Accordingly, the purpose of this write-up is to suggest a systematic approach for the development of a validated simple, sensitive, and stability indicating RP-HPLC method that should meet the current ICH and regulatory requirements [22].

## 2. Experimental

The present method was designed to be easy to use, sensitive, and rapid. Separation and quantification of PG and TMP in pharmaceutical drug formulations and blood were achieved with an isocratic elution and with dual wavelength technique.

### 2.1. Apparatus and Materials

SIL 10A autoinjector HPLC system comprising of SCL 10A system, controller, SPD 20A prominence UV/VIS detector, and Shimadzu LC 20 AT pump with LC Solutions software, was used. Separation was performed on a Hibar *μ*Bondapak ODS C_18_ HPLC column (4.6 × 250 mm; 10 *μ*m bead size) and maintained at 25°C. A UV-visible Shimadzu 1650 PC spectrophotometer with UV Probe software, ultrasonic cleaner (Elmasoni E 60 H), Jenway 3240 pH meter, and Sartorius TE2145 analytical balance were used in the research work. Throughout the work only amber glass flasks were used to avoid light effect on the solution of PG and TMP standards and samples.

Trimethylphloroglucinol and phloroglucinol were kind gifts from a National Pharmaceutical company, and sulphuric acid (Merck, Germany) and acetonitrile (HPLC grade) were purchased from Fisher Scientific. The injectable containing PG and TMP were obtained from commercial source (SPASFON Injection, SPADIX Injection, FUROSINOL Injection, and ANAFORTAN PLUS Injection) labeled to contain 10 mg/mL and 0.01 mg/mL of PG and TMP, respectively. Distilled water was procured through RO plant (Waterman, Pakistan).

### 2.2. Chromatographic Conditions

The HPLC analysis was carried out at ambient temperature. The compound was chromatographed isocratically with a mobile phase consisting of acetonitrile (HPLC grade): sodium n-heptane sulphonate (0.005 M)  : sulfuric acid 0.1 M (50 : 50 : 0.3,  v/v/v) with the pH adjusted if required to 3.0 ± 0.1 using 0.1 M sulfuric acid or 0.1 M NaOH. While for sample and standard preparation diluent was prepared from acetonitrile (HPLC grade): distilled water (50 : 50, v/v%). The mobile phase was filtered by passing through a 0.45 *µ*m membrane filter (Millipore, Bedford, MA, USA) and degassed via sonication. The flow rate was 1.0 mL/min, and the injected volume was 20 *µ*L. The effluent was monitored at dual wavelength of 266 and 205 nm.

## 3. Analytical Procedure 

### 3.1. Standard Preparation

In a 100 mL volumetric flask, about 10 mg of TMP reference standard was weighed accurately and dissolved in diluent to have a stock solution of 100 *µ*g/mL. Similarly in another 100 mL volumetric flask, accurately 500 mg of PG reference standard was weighed and dissolved in diluent, sonicated for 2 minutes, and let to cool to room temperature. Then, 5 mL of TMP stock solution was added, and volume was made up to the mark with the same diluent. Finally 2 mL of this solution was diluted in 100 mL of diluent to get a 100 *µ*g/mL of PG and 0.1 *µ*g/mL of TMP working standard solution. The standard was then filtered through 0.45 *µ*m filter paper and injected into the HPLC system.

### 3.2. Sample Preparation of Injectable Solutions

For making a sample of 100 *µ*g/mL of PG and 0.1 *µ*g/mL of TMP, 10 ampoules were broken, and content was mixed to get an evenly homogenized stock sample. The sample volume was taken accurately equivalent to 10 mg of PG & 0.01 mg of TMP in 100 mL volumetric flask, and 50 mL of diluent was added. The sample was sonicated for 1 minute and then diluent was added up to the mark and placed on stirrer for 5 minutes. The sample was then filtered through 0.45 *µ*m filter paper and injected into the HPLC system.

### 3.3. Sample Preparation of Serum

Blood samples were collected from healthy volunteers in evacuated glass tube through an indwelling cannula placed on forearm vein by a trained clinical laboratory technician. The volunteers were not involved in any medication, smoking, and strenuous activity. The blood was shaken and centrifuged at 6,000 rpm for 30 min to separate out plasma. 9 mL acetonitrile was added to 1 mL plasma and centrifuged at 6,000 rpm for 30 min to deproteinate it. The supernatant serum thus obtained was stored at 20°C and filtered for subsequent analysis. For making a working sample solution of 12.5 mL, stock sample was taken in 50 mL flask followed by 30 mL of serum addition. The sample thus obtained was stirred for 10 minutes and then diluent was added up to the mark. Further 2 mL of this solution was diluted in 50 mL of flask with the aid of diluent. All samples prepared were filtered through 0.45 *µ*m membrane filter and injected in triplicate into the HPLC system.

### 3.4. Sample Preparation for Degradation Studies

For this purpose 5 mL of the stock sample was diluted in 100 mL diluent to make a stock sample solution. For working purpose, 10 mL of stock sample solution was diluted in four individual 50 mL volumetric flasks, and 15 mL of degrading agent were added to each flask, with the exception of one to which only diluent was added; these included 0.1 N HCl, 0.1 N NaOH, and 30% H_2_O_2_, and then to each flask diluent was added up to the mark. All the four samples were placed in water bath at 60°C for one hour. The samples were then filtered through 0.45 *µ*m membrane filter and injected into the HPLC system. Intentional degradation was attempted with stress conditions exposing the drugs to acid (0.1 N HCl), alkali (0.1 N NaOH), hydrogen peroxide (30%), and heat (60°C) to evaluate the ability of the proposed method to separate drugs from its degradation products. For all conditions the temperature was kept constant at 60°C for a period of one hour.

### 3.5. Stability Studies

For stability studies the commercially available injection (parenteral) samples in ampoules were placed at accelerated conditions of temperature that is at 40°C with 75% relative humidity and at ambient conditions of 30°C temperature with 65% relative humidity in environmental chamber for six months. The stability protocol in Table 6 was followed for six months.

### 3.6. Method Validation

ICH guidelines [22] were used to perform method validation studies. Various procedures including specificity, linearity, range, accuracy, and intraday and interday precision were evaluated.

To study linearity, twenty dilutions of standard were made to prepare standard solution in range from 10% to 200%, that is, from 10 *µ*g/mL to 200 *µ*g/mL of PG and from 0.01 *µ*g/mL to 0.2 *µ*g/mL of TMP, respectively, drugs content. The standard calibration curve was generated using regression analysis with online help (http://wessa.net/). For specificity commonly used excipients in injection preparation were spiked in a preweighed quantity of drugs to determine the effect and interference of excipients in quantification of the drugs.

In order to find out the repeatability and reproducibility of the method, precision was studied to find out intra- and interday variations in the test method of PG and TMP in the concentration range of 80–120 *μ*g/mL for PG and 0.08–0.12 *μ*g/mL for TMP, respectively. Precision was determined by analyzing corresponding bulk sample daily for a period of three days and three times a day with an interval of 8 hours against a freshly prepared standard. For determining accuracy the PG and TMP reference standard were accurately weighed and spiked to the injection sample at three different concentration levels to gain 110%, 120%, and 130% of both APIs. At each level, samples were prepared in triplicate, and the recovery percentage was determined.

Limit of detection and quantification (LOD and LOQ) for the method were established by sequentially diluting the standard solutions at decreasing concentrations, in the range of 100–1 pg/mL for PG and 10–1 pg/mL for TMP. The limit of detection was defined as the concentration for which a signal-to-noise ratio of 3 was obtained, and for quantification limit, a signal-to-noise ratio of 10 was considered. The standards were injected in LC system, and measured signals from the diluted standards were compared with those of blank samples.

To study robustness, samples of injections were assayed with deliberate variation in the method parameters, such as in the chromatographic conditions, like mobile phase, flow rate, and temperature so forth. Justification of system suitability was established by calculating % relative standard deviation of replicate injections and analyzing the symmetry, resolution of the standard peaks, and theoretical plates of the column.

## 4. Results and Discussion

The HPLC method development and its validation are the prioritized requirements for any drug available in the market to ensure the quality of the products. A few methods are available for determination of the APIs as described earlier, but many of them are used only for certain definite objectives and lack generalization for simultaneous analytical applications in form of pharmaceutical products and serum. Similarly none of them are as much sensitive as ours in terms of their precision, accuracy, limit of detection (LOD), and limit of quantification (LOQ) especially as compared to [7, 9, 12, 14] whose LOD or LOQ was in microgram range only, while ours method is sensitive enough to be used for pharmacokinetic studies. Here, the LOD and LOQ for both APIs are 10 pg/mL and 33 pg/mL, respectively; however considering their ratio in sample formulation, the LOD and LOQ for PG are taken as 10 ng/mL and 33 ng/mL, respectively, and hence LOQ and LOD for TMP can also be considered as the levels of sensitivity of the method.

### 4.1. Method Development and Optimization

**Figure 2:**
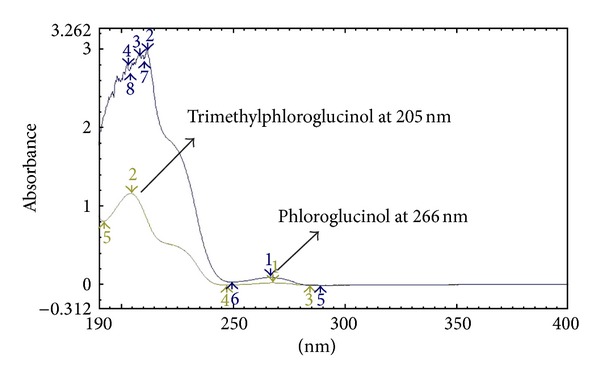
UV spectrum of PG (100 *µ*g/mL) and TMP (0.1 *µ*g mL^−1^).

For developing an efficient method for the simultaneous analysis of PG and TMP, parameters such as detection wavelength, mobile phase composition, optimum pH, and concentration of the standard solutions were comprehensively studied. Both PG and TMP were diluted in dilution solvent and then run through UV spectrophotometer in UV range of 190 nm–400 nm to get maximal wavelengths, Figure 2, where maximum absorbance was gained, that is, 266 nm for both APIs.

However considering the difference of concentration in both APIs that is 10 to 0.01 mg/mL for PG and TMP, respectively, therefore 205 nm was used for TMP and 266 nm for PG.

Mobile phase was selected in terms of its components and their proportions. The chromatographic parameters were evaluated using a Hibar *μ*Bondapak ODS C_18_ column the mobile phase composed of acetonitrile : buffer of given proportion promoted a short run time (10 min) without any interference, so this condition was adopted in subsequent analysis.

The literature survey also revealed that almost all the methods developed so far have utilized acetonitrile as a major component in mobile phases. Acetonitrile is always preferred due to the supreme solubility properties and UV absorbance characteristics, and there is no counterpart substitute for acetonitrile in the reverse phase HPLC and UV application. Therefore, keeping in view the chromatography type and the detection wavelengths in use, acetonitrile was chosen for analysis, though in our previous work [21] we used methanol for its cost effectiveness.

### 4.2. Validation Studies

The linearity ranges were found to be 10–200 *µ*g/mL for PG and 0.01–0.2 *µ*g/mL for TMP. The assay was judged to be linear, as the correlation coefficient was 0.9999 and 0.9992 for PG and TMP, respectively, as calculated by the least-square method. A linear correlation was found between the peak areas and the concentrations of APIs, in the assayed range. The regression analysis data are presented in Table 1.

**Figure 3:**
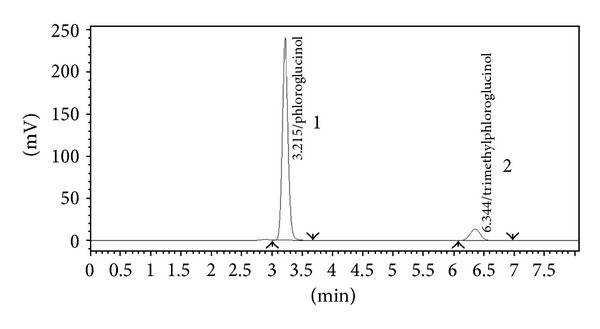
Representative chromatogram of sample.

Chromatogram shown in Figure 3 proves specificity or selectivity of the assayed method, as the chromatograms in samples were found identical with standard chromatogram and no interference peak was observed in sample chromatogram. Peak purities higher than 98.0% were obtained in the chromatograms of sample solutions, demonstrating that other compounds did not coelute with the major peaks. The chromatogram obtained with the mixture of the injection excipients proves that there is no interference from excipients and peak of interest that fulfills all the requirements of symmetrical peak, and hence, the specificity is confirmed.

The precision of an analytical method is the degree of agreement among individual test results when the method is applied repeatedly to multiple sampling of homogeneous bulk. Intraday precision of the method was evaluated at three different independent concentrations that are, 80%, 100%, and 120% for both drugs (*n* = 3) by determining their assays. Interday precision of the method was tested for 3 days at the same concentration levels. Solutions for calibration curves were prepared every day on fresh basis. Since the interday and intraday precision obtained %RSD were less than 2% it is assured that the proposed method is quite precise and reproducible, as shown in Table 2.

The accuracy was investigated by spiking reference standards to a mixture of the injection excipients at three different concentration levels, that is, multiple level recovery studies, and subjected to the proposed HPLC method. The obtained recovery (*n* = 9) was 98.86–100.96% (RSD% = 0.488) for PG and 98.56%–101.24% (RSD% = 0.139) for TMP, demonstrating the accuracy of the method. Percentage recoveries for marketed products were found to be within the limits, Table 3.

The statistical analysis showed no significant difference between results obtained by employing the analytical conditions established for the method and those obtained in the experiments in which variations of some parameters were introduced. The parameters used in system suitability test were symmetry of peaks, tailing factor, resolution, and RSD% of peak area for replicate injections. Thus, the method showed to be robust for changes in mobile phase acetonitrile proportion, mobile phase pH, flow rate, and column temperature, Table 4.

### 4.3. Degradation Studies

During the degradation study, it was observed that upon treatment of PG and TMP with base (0.1 M NaOH), acid (0.1 M HCl), hydrogen peroxide (30%), and heat, maximum degradation was observed in acid and H_2_O_2_.  Table 5 shows the extent of degradation of both drugs under various stress conditions.

**Figure 4:**
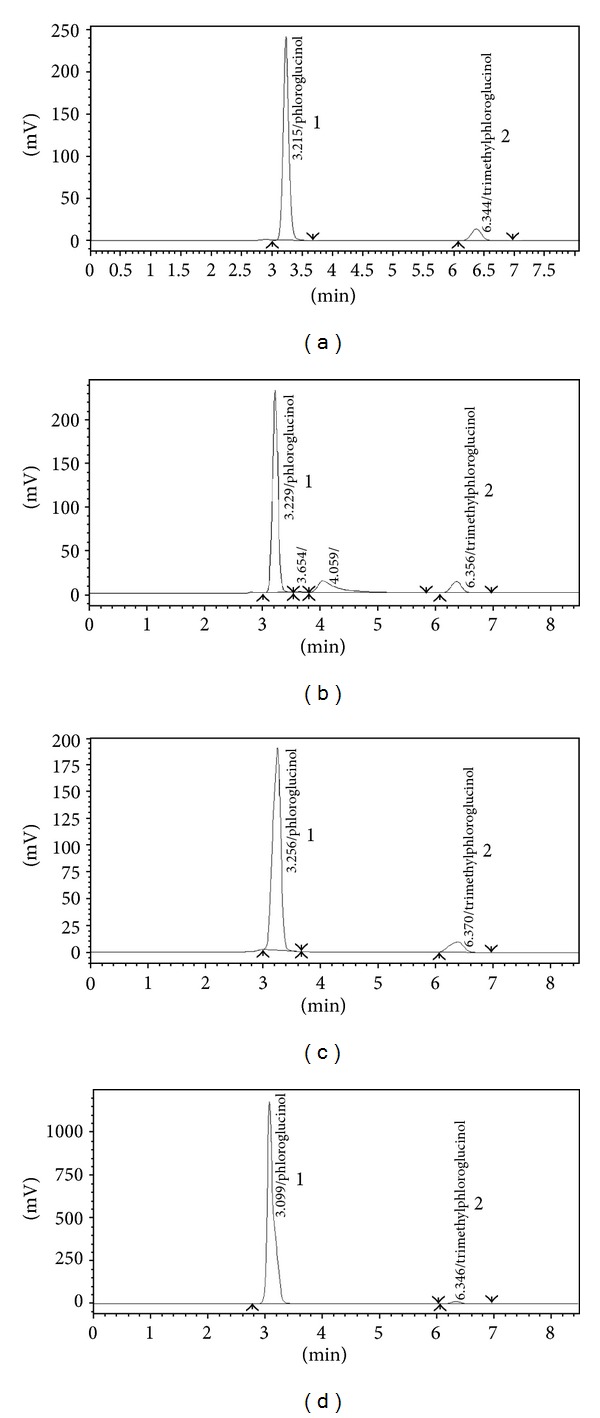
Representative chromatograms of phloroglucinol (1), trimethylphloroglucinol (2), and forced degradation, heat treated sample (a), acid treated sample (b), base treated sample (c), and H_2_O_2_ treated Sample (d).

It is a fact that phenolic compounds, such as phloroglucinol, are known to undergo ready oxidation in basic solutions (due to dissolved oxygen) and by agents such as hydrogen peroxide which increases the absorbance capacity of the molecules, causing increased peak area and hence % calculated amount. And the same is revealed by the data given that PG is more vulnerable to stress conditions especially alkaline and oxidation treatments as compared to TMP. Further, Figures 4(a)–4(d) show the chromatograms of forced degraded samples. Degraded peaks are observed in case of acid hydrolysis, and peak had been broadened due to extra absorbance caused by oxidation of the PG, whereas no appreciable degradation was observed in heat treated sample; hence the drug is stable under heat stress conditions.

### 4.4. Stability Studies

Stability testing is an important part of the process of drug product development. The purpose of stability testing is to provide evidence of how the quality of a drug substance or drug product varies with time under a variety of environmental conditions, like temperature, humidity, and light and enable recommendation of storage conditions, retest periods, and shelf life to be established. The two main aspects of drug product that play an important role in shelf-life determination are assay of the active drug and the degradation products generated during stability studies.

The proposed assay method was applied to the stability study of commercially available injections, for which the samples were placed at 30°C with relative humidity of 65% and at 40°C with relative humidity of 75%. Stability study was performed according to stability protocol as described in the previous section. Samples were analyzed and percentage of contents was measured, Table 6. According to the results obtained both APIs were found to be stable at applied conditions of temperature and relative humidity and were accurately analyzed with the proposed method.

## 5. Conclusions

The proposed new HPLC method described in this paper provides a simple, universal, convenient, and reproducible approach for the simultaneous identification and quantification of phloroglucinol and trimethylphloroglucinol in human serum and pharmaceutical formulations (injectable) with good separation and resolution. In addition, this method has the potential application to clinical research of drug combination. Analytical results are accurate and precise with good recovery and lowest detection limit values. In short, the developed method is simple, sensitive, easy, and efficient having short chromatographic time and can be used for routine analysis in QC laboratory and therapeutic monitoring.

## Figures and Tables

**Table 1:** Calibration curve data and validation parameters.

Parameter	Inference	
	Phloroglucinol	Trimethylphloroglucinol
Linearity range (*µ*g/mL)	10–200	0.01–0.2
Correlation coefficient (*r*)	0.9999	0.9992
Regression equation (*y* = *mx* + *c*) Slope (*m*)	402.66	3.12
Intercept (*c*)	0	0
For commercial formulation		
Limit of detection (LOD) (ng/mL)	10	0.01
Limit of quantification (LOQ) (ng/mL)	33	0.033
For method sensitivity		
Limit of detection (LOD) (pg/mL)	10	0.01
Limit of quantification (LOQ) (pg/mL)	33	0.033

**Table 2:** Interday and intraday precision and recovery studies.

Active drugs	PG	TMP	PG	TMP	PG	TMP	Interday RSD%*	
Nominal concentration (*µ*g/mL)	80	0.08	100	0.1	120	0.12	PG	TMP
Day 1	99.74	100.29	100.65	98.56	100.28	100.72	0.456	1.146
Day 2	100.38	98.92	100.96	101.24	99.92	99.78	0.521	1.172
Day 3	98.86	100.79	99.99	100.62	98.99	99.71	0.628	0.579
Mean	**99.66**	**100.34**	**100.53**	**100.14**	**99.73**	**100.1**	0.488	0.139
Intraday RSD%	0.76	0.97	0.49	1.4	0.66	0.56		

*RSD%: relative standard deviation (should be less than 2.0).

All results are expressed in percentage values.

**Table 3:** Contents of PG and TMP in the fixed dose combination injections.

Sample injection	Content (%) ± S.D.	
	PG	TMP
SPASFON	99.75 ± 0.19	98.72 ± 0.43
SPADIX	98.59 ± 0.81	97.97 ± 0.71
FUROSINOL	100.19 ± 0.53	100.59 ± 0.19
ANAFORTAN PLUS	99.41 ± 0.15	97.18 ± 0.38

S.D.: standard deviation.

**Table 4:** Robustness of the method.

Chromatographic conditions	Variation	Retention time (minutes)	
		PG	TMP
Temperature (°C)	23	3.099	6.346
	27	3.217	6.381

Flow rate (mL/min.)	0.9	3.341	6.413
	1.1	2.89	5.994

Vol. of acetonitrile (%)	48	3.397	6.499
	52	2.982	5.619

**Table 5:** Summary of forced degradation results.

Stress conditions	Time (h)	Assay of active substance (%)		Increase due to oxidation (%)	
		PG (100 *µ*g/mL)	TMP (0.1 *µ*g/mL)	PG (100 *µ*g/mL)	TMP (0.1 *µ*g/mL)
Acid hydrolysis (0.1 M HCl)	1	92.88	96.46	7.12	3.54
Base hydrolysis (0.5 M NaOH)	1	129.17	101.09	29.17	1.09
Oxidation (30% H_2_O_2_)	1	623.16	103.14	523.16	3.14
Thermal (60°C)	1	106.53	105.94	6.53	5.94

**Table 6:** Summary of stability studies.

Test (claimed content)	Interval								
	Initial	1 month	2 months	3 months	4 months	5 months	6 months	Mean	RSD%
Studies at accelerated (40°C + 75% H)									
PG (10 mg/mL)	100.35	99.96	99.47	99.26	98.97	98.43	98.15	99.04	0.79
TMP (0.01 mg/mL)	99.85	98.98	98.41	98.13	97.97	97.86	97.53	98.15	0.80

Stability studies at long term (30°C + 65% H)									
PG (10 mg/mL)	100.35	100.13	100.38	100.1	99.85	99.23	99.47	99.87	0.44
TMP (0.01 mg/mL)	99.85	99.62	99.39	98.93	98.91	98.37	98.12	98.89	0.64

All results are expressed in percentage values.
